# Synthesis of (3*R*)-acetoin and 2,3-butanediol isomers by metabolically engineered *Lactococcus lactis*

**DOI:** 10.1038/srep36769

**Published:** 2016-11-18

**Authors:** Vijayalakshmi Kandasamy, Jianming Liu, Shruti Harnal Dantoft, Christian Solem, Peter Ruhdal Jensen

**Affiliations:** 1 National Food Institute, Technical University of Denmark, DK-2800 Kgs. Lyngby, Denmark

## Abstract

The potential that lies in harnessing the chemical synthesis capabilities inherent in living organisms is immense. Here we demonstrate how the biosynthetic machinery of *Lactococcus lactis*, can be diverted to make (3*R*)-acetoin and the derived 2,3-butanediol isomers *meso*-(2,3)-butanediol (m-BDO) and (2*R*,3*R*)-butanediol (R-BDO). Efficient production of (3*R*)-acetoin was accomplished using a strain where the competing lactate, acetate and ethanol forming pathways had been blocked. By introducing different alcohol dehydrogenases into this strain, either EcBDH from *Enterobacter cloacae* or SadB from *Achromobacter xylosooxidans*, it was possible to achieve high-yield production of m-BDO or R-BDO respectively. To achieve biosustainable production of these chemicals from dairy waste, we transformed the above strains with the lactose plasmid pLP712. This enabled efficient production of (3*R*)-acetoin, m-BDO and R-BDO from processed whey waste, with titers of 27, 51, and 32 g/L respectively. The corresponding yields obtained were 0.42, 0.47 and 0.40 g/g lactose, which is 82%, 89%, and 76% of maximum theoretical yield respectively. These results clearly demonstrate that *L. lactis* is an excellent choice as a cell factory for transforming lactose containing dairy waste into value added chemicals.

Acetoin and 2,3-butanediol (2,3-BDO) are valuable bio-based chemicals that have a wide range of applications. Acetoin is an important flavor compound, which is found naturally in various foods and is commonly used as a food additive. It also serves as a building block for the synthesis of cosmetics, pharmaceuticals and various chemicals^1^,^2^. Like acetoin, 2,3-BDO is a versatile chemical with many applications, e.g. it is used as a component in perfumes and fumigants, for making synthetic rubber, pharmaceuticals and plastics, and it is also considered to be an excellent biofuel^2^,^3^.

Acetoin and 2,3-BDO can be formed in few steps from pyruvate, where the first step is the decarboxylative condensation of two pyruvate molecules into α-acetolactate, which is a reaction catalyzed by α-acetolactate synthase (Als). One possible fate for α-acetolactate is to be converted into (3*R*)-acetoin, a reaction catalyzed by the enzyme α-acetolactate decarboxylase (Ald). (3*R*)-acetoin can be reduced into either (2*R*,3*R*)-butanendiol (R-BDO) or *meso*-(2,3)-butanediol (m-BDO) by specific 2,3-butanediol dehydrogenases (BDHs) (Fig. 1). Alternatively, α-acetolactate, because of its unstable nature, spontaneously can decompose into diacetyl through oxidative decarboxylation. Diacetyl can subsequently be reduced into (3*S*)-acetoin, where (3*S*)-acetoin can give rise to either m-BDO or (2*S*, 3*S*)-butanediol (S-BDO), again depending on the type of BDHs involved (Fig. S1). In addition to the dedicated BDHs, there are also certain secondary alcohol dehydrogenases (sADH) that are able to accept 2,3-BDO as a substrate^4^,^5^,^6^,^7^,^8^,^9^. Specifically sADH from *Clostridium beijerinckii* and *Thermoanaerobacter brockii* have been shown to convert (3*R*)-acetoin into R-BDO^9^.

There are several native producers of 2,3-BDO, e.g. *Klebsiella oxytoca* and *Klebsiella pneumoniae*. However these organisms are not considered to be safe, and another disadvantage could be that mixtures of the various 2,3-BDO isomers are produced. The pure isomers sometimes have useful properties or specific applications, which make them very valuable, e.g. aqueous solutions of the stereoisomers of 2,3-butanediol have very low freezing points, as low as −65 °C, and they could therefore be used as antifreeze agents^10^. Another important application for stereoisomers is as chiral building blocks for making drugs^10^,^11^,^12^. Despite the many reports describing efficient production of 2,3-BDO by native and engineered microorganisms, current production is still based on chemical synthesis, indicating that microbial production is still not sufficiently cost efficient. One way to address this could be to base production on low-value waste streams rich in carbohydrates. Whey derived waste from the dairy industry is a good example, and it is generated in large amounts. We have recently demonstrated that the lactic acid bacterium *Lactococcus lactis* is well-suited for converting lactose-rich dairy waste into bioethanol^13^. *L. lactis* is a safe microbe used extensively in the dairy industry, especially for making cheese, and this lactic acid bacterium already possesses the metabolic pathway for producing (3*R*)-acetoin^14^. *L. lactis* has been engineered into producing a broad range of other useful compounds and shows great general potential as a cell factory for several reasons: It has a high glycolytic flux, is able to metabolize most of the common sugars, has a well-characterized metabolic network and is simple to manipulate genetically^15^,^16^.

In this study, we engineer *L. lactis* into producing (3*R*)-acetoin and the two 2,3-BDO stereoisomers m-BDO and R-BDO. First we demonstrate efficient (3*R*)-acetoin formation using an *L. lactis* strain where all the major by-product pathways involving pyruvate have been eliminated. We subsequently introduce codon-optimized versions of the robust butanediol dehydrogenase (EcBDH) from *E. cloacae* and the alcohol dehydrogenase (SadB) from *A. xylosooxidans*^17^, which leads to the production of m-BDO and R-BDO respectively. Finally, we show the efficient production of these chemicals from processed whey waste and thus demonstrate that it is feasible to develop sustainable bioprocesses based on *L. lactis*.

## Results

### Tolerance of *L. lactis* to acetoin and 2,3-butanediol

When selecting a candidate organism for bio-production, it is relevant to determine the tolerance towards the compound produced. Acetoin has previously been shown to be toxic to bacteria^18^ and so has 2,3-BDO, although 2,3-BDO is less toxic than other alcohols^12^,^19^. When compared to other microbes, lactic acid bacteria in general are quite tolerant to organic acids and alcohols like butanol and ethanol^20^,^21^, and thus, this group of organisms fulfills an important success criterion within bio-based production.

In this study we found only a slight effect on growth from having 20 g/L acetoin or 2,3-BDO present during anaerobic growth (Table 1). In general acetoin or 2,3BDO toxicity on cell growth was more pronounced during aerobic conditions. For 2,3-BDO, as expected, there was a direct correlation between concentration and the effect on growth, and at the highest concentration tested (100 g/L) the specific growth rate was reduced by 64% and 78% during aerobic and anaerobic cultivation respectively when compared to the control without any added 2,3-BDO. Acetoin appeared to be more toxic than 2,3-BDO, and at 60 g/L the growth rate was reduced by almost 80 and 96% respectively and growth almost ceased at 80 g/L.

### Construction of an *L. lactis* platform for enantiopure (3*R*)-acetoin production

For most wild type strains of *L. lactis*, including the one we use in this study (MG1363), lactate is the dominant fermentation product when growing on readily fermentable sugars^13^, which enables the regeneration of the NAD^+^ consumed in glycolysis. When lactate formation is prevented, e.g. through inactivation of the lactate dehydrogenase activity, *L. lactis* is able to maintain the redox balance by changing product formation, and the outcome is usually formate, ethanol and acetate, as well as smaller amounts of other products such as m-BDO^13^. To fully redirect the flux towards (3*R*)-acetoin and 2,3-BDO, it is necessary to inactivate all the competing pathways at the pyruvate node and as a starting point we used our previously constructed strain CS4363 where genes encoding three lactate dehydrogenase (LDH) homologues (*ldh*, *ldhB*, *ldhX*), phosphotransacetylase (PTA) and alcohol dehydrogenase (ADHE) had been deleted^20^. This strain had lost its ability to grow under anaerobic conditions since all the pathways for NAD^+^ regeneration were inactive. Under aerobic conditions, however, the water-forming NADH oxidase (NOX) can regenerate NAD^+^ and thus allow for growth. When this strain was grown under aerobic conditions, the endogenous activities of Als and Ald, efficiently ensured that pyruvate was transformed into mainly (3*R*)-acetoin (Table 2).

*L. lactis* has two genes encoding butanediol dehydrogenases namely *butB* and *butA*. It has previously been shown that when *butA* was over-expressed in an engineered *L. lactis* strain, m-BDO could be formed^22^. To completely eliminate m-BDO formation and achieve (3*R*)-acetoin as the sole fermentation product, we therefore further inactivated the native *butB* and *butA* genes, which are co-located in an operon, thus generating VJ017 (MG1363 Δ^*3*^*ldh* Δ*pta* Δ*adhE* Δ*butBA*) (Table 3). This strain had a slightly higher yield of (3*R*)-acetoin when compared to CS4363.

**Figure 2:**
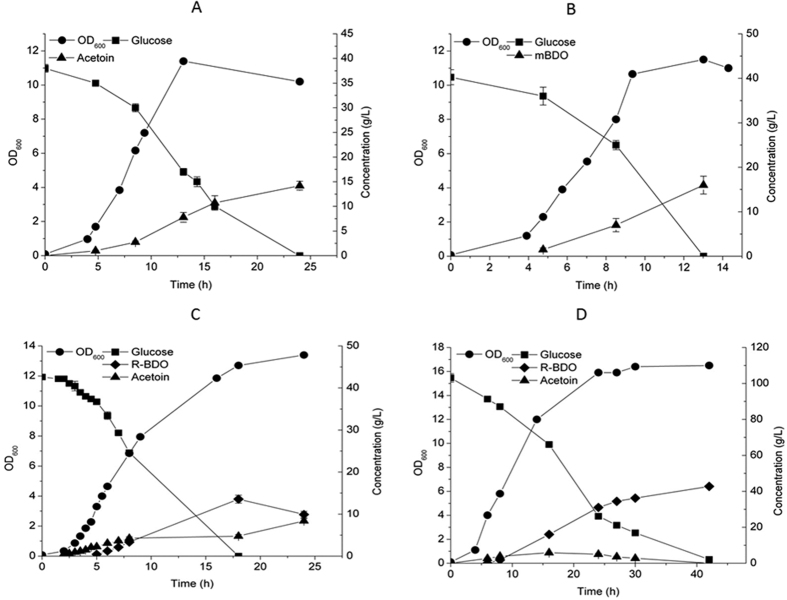
(3*R*)-acetoin and 2,3-BDO isomer synthesis in engineered *L. lactis* strains. The engineered *L. lactis* strains, VJ017, VJ018 and mBD001 were grown in M17 medium under aerobic conditions supplemented with glucose. (**A**) VJ017, (**B**) mBD001, (**C**) VJ018 with 40 g/L initial glucose and (**D**) VJ018 with 103 g/L initial glucose. Error bars represent standard deviation.

We found that the recombinant strain VJ017, when grown in aerobic conditions in shake flask with M17 medium supplemented with around 40 g/L glucose, could synthesize 14 g/L of enantiopure (3*R*)-acetoin, i.e. an enantiomeric excess (e.e.) of 100%, in 24 h with yield of 0.37 g/g glucose which is 76% of theoretical maximum (Fig. 2A).

### Extending the metabolic pathway from (3*R*)-acetoin to m-BDO

As shown above, the main fermentation product of VJ017 was (3*R*)-acetoin. Since (3*R*)-acetoin is a precursor for m-BDO we decided to introduce a 2,3-butanediol dehydrogenase from *E. cloacea*, EcBDH, which has been reported to be highly efficient at reducing acetoin into m-BDO and which in addition is an extremely robust enzyme^23^. We transformed VJ017 with our previously generated vector, pJM001^24^ containing EcBDH driven by high strength synthetic promoter to generate mBD001. We found that mBD001 was able to synthesize m-BDO (sole isomer detected) with a high yield (Table 2). Within 13 h, mBD001 could consume 40 g/L of glucose, and (3*R*)-acetoin was not detected in the medium, which demonstrated that the conversion from (3*R*)-acetoin to m-BDO indeed was efficient. The strain produced 16 g/L m-BDO in 13 h with a yield of 0.4 g/g glucose which is 80% of theoretical maximum (Fig. 2B). When the fermentation was prolonged after glucose exhaustion, the m-BDO titer decreased to 14 g/L with formation of 1.46 g/L (3*R*)-acetoin between 13 h and 18 h, and this was due to the reversibility of the (3*R*)-acetoin to m-BDO reaction.

### Extending the metabolic pathway from (3*R*)-acetoin to enantiopure R-BDO

(3*R*)-acetoin is also the precursor of another 2,3-BDO isomer, namely R-BDO. Several specific R-BDO forming 2,3-butanediol dehydrogenases have been reported previously^12^,^25^,^26^, but as mentioned in the introduction there are secondary alcohol dehydrogenases (sADH) that can carry out this transformation just as efficiently^10^. We used an sADH from *A. xylosooxidans*, which we first expressed in the wild type (VJ021 = MG1363 expressing SadB). Crude enzyme extracts of VJ021 were tested for activity towards R-BDO, and where the control (crude extract of MG1363) did not display any activity, the extract of VJ021 rapidly oxidized R-BDO in the presence of NAD^+^ (data not shown).

Next, we expressed SadB in the acetoin-producing platform VJ017, and obtained the strain VJ018, which was able to produce R-BDO at high yields (Table 2). HPLC analysis was used to differentiate between the meso-form and the R-form of BDO, and it was clear that R-BDO was the product when (3*R*)-acetoin was used as substrate. Formation of S-BDO could be ruled out, since this isomer is formed from diacetyl^24^, which we also substantiated by GC analysis (Fig. S3). We found that VJ018 could consume 40 g/L glucose within 18 h and produce 14 g/L of enantiopure R-BDO (Fig. 2C). During the first 8 h we detected (3*R*)-acetoin formation, which indicated that there was an imbalance in the cofactor-partitioning between NOX and SadB.

Interestingly, after substrate exhaustion, the strain oxidized some of the R-BDO formed back into (3*R*)-acetoin, thus the R-BDO titer decreased from 14 g/L to 10 g/L (between 18 h and 24 h). To avoid R-BDO oxidation we increased the initial glucose concentration to 103 g/L, and interestingly the (3*R*)-acetoin produced in the initial growth phase (around 6 g/L) was reduced into R-BDO in the later phase (Fig. 2D). In this case more glucose was consumed during the growth phase, while the glucose consumption rate decreased during the stationary phase, and 43 g/L R-BDO was obtained by 42 h with a yield of 0.41 g/g glucose, which corresponds to 83% of theoretical maximum (Fig. 2D).

### (3*R*)-Acetoin and 2,3-BDO isomers production using whey waste

**Figure 3:**
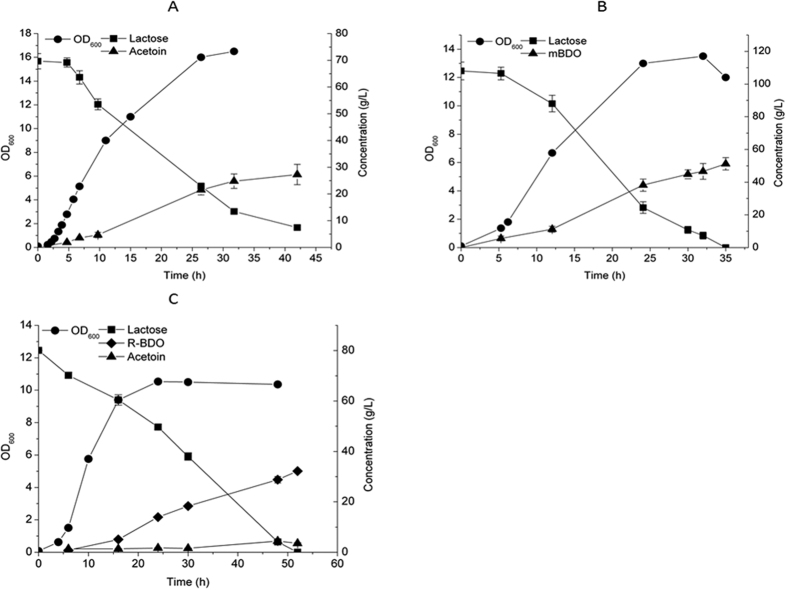
(3*R*)-acetoin, m-BDO and R-BDO production using residual waste stream whey permeate. The recombinant strains AL002, mL001 and VJ031 were grown in diluted RWP with 2% yeast extract. (**A**) AL002, (**B**) mL001 and (**C**) VJ031. Errors bars represent standard deviation.

The strains used in this study, for producing (3*R*)-acetoin (VJ017) and 2,3-BDO (mBD001 and VJ018), are derived from the laboratory strain MG1363, a derivative of the dairy strain NCDO712 which cannot use lactose as sole carbon source. To enable production of these compounds from lactose containing whey waste, we therefore re-introduced the lactococcal plasmid-pLP712 (55.395 kbp) derived from NCDO712, which carries all the genes needed for metabolizing lactose, including *lacEF, lacG* and *lacABCD*, into VJ017, VJ018 and mBD001 to generate AL002, VJ031 and mL001 respectively. The strains transformed with this plasmid were all able to grow in defined medium (SAL) containing lactose as sole carbon source. Residual whey permeate (RWP), which is a lactose rich side stream generated when Arla Foods Ingredients Group P/S (http://www.arlafoodsingredients.com/) processes whey permeate, was subsequently tested as a feedstock. RWP contains around 15% lactose but has an insufficient level of amino acids to support growth^13^. As detailed in Materials and Methods we supplemented this feedstock with yeast extract as a nitrogen source and characterized growth and production of the (3*R*)-acetoin and BDO-producing strains in this medium. AL002 produced 27 g/L (3*R*)-acetoin in 42 h (Fig. 3A) from an initial lactose concentration of 70 g/L and around 6 g/L lactose was left unconsumed even after 72 h. The productivity was 0.64 g/L·h and the yield was 82% of the theoretical yield. In contrast, mL001 consumed 108 g/L lactose and produced a high titre of 51 g/L m-BDO with the yield and volumetric productivity of 0.47 g/g and 1.46 g/L·h respectively and the yield obtained was 89% of theoretical maximum (Fig. 3B). Similar to what was observed when M17 medium was applied, mL001 produced m-BDO as the sole product and (3*R*)-acetoin was not detected during throughout the fermentation. For VJ018, large amounts of (3*R*)-acetoin were formed in addition to R-BDO (equimolar amounts) when using RWP under the same conditions used for mL001 (data not shown). Although the reason is unclear, this could be due to a higher NOX activity in the RWP medium than in the M17 medium used previously. It is well-known that NOX activity can vary with the aeration level^27^,^28^, and for this reason we tried to limit aeration by changing flask to medium ratio to 2.5 : 1 (100 ml medium in 250 ml flask). We used two different initial lactose concentrations 80 and 110 g/L, and observed that the final biomass achieved was 27% lower at the high lactose concentration and also the lactose consumption rate was affected negatively. With initial 80 g/L lactose, the R-BDO titer reached 32 g/L in 52 h with the yield of 0.4 g/g lactose (76% of theoretical maximum) with the productivity of 0.62 g/L·h (Fig. 3C). The strain also formed (3*R*)-acetoin to a final titer of 3.5 g/L with a yield of 0.044 g/g lactose, which was much lower than when using higher flask volume to medium ratio (8.5:1) where the (3*R*)-acetoin yield was around 0.2 g/g lactose.

## Discussion

In the present study, we used a metabolically engineered *L. lactis* strain, VJ017 (MG1363 Δ^*3*^*ldh* Δ*pta* Δ*adhE* Δ*butBA*) for making (3*R*)-acetoin, and harness the intracellular NOX activity for regenerating NAD^+^. A similar strategy has been used where NOX from *L. lactis* was heterologously expressed in other bacteria to provide a redox sink and improve production of chemicals like pyruvate, acetoin and 2,3-BDO^29^,^30^,^31^.

We also extended the pathway to produce the two 2,3-butanediol isomers m-BDO and R-BDO using EcBDH from *E. cloacae* and the secondary activity of the alcohol dehydrogenase SadB from *A. xylosooxidans* respectively. Formation of the specific isomers of 2,3-BDO involves the use of specific BDHs (butanediol dehydrogenases) and which isomers that are formed also depends on the stereoisomeric form of acetoin^32^,^33^,^34^,^35^. (3*R*)-acetoin can be converted to either m-BDO or R-BDO and (3*S*)-acetoin can be converted to m-BDO and S-BDO. The enzymes characterized as (2*R*,3*R*)-BDHs from *P. polymyxa*, and *B. subtilis* can convert (3*R*)-acetoin and (3*S*)-acetoin to R-BDO and m-BDO respectively^10^,^36^. In the present study, when we expressed SadB in *L. lactis* the outcome was R-BDO and formation of R-BDO is in line with previous work where secondary alcohol dehydrogenases have been found to catalyze formation of this isomer^9^.

Normally when 2,3-BDO is formed from glucose, only one of the two NADH generated in glycolysis is consumed, thus creating an excess of one NADH. Since *L. lactis* harbors a cytosolic NOX activity it is possible to regenerate NAD^+^ by using aerobic growth conditions. The 2,3-BDO forming strains VJ018 and mBD001 consumed glucose more efficiently than the (3*R*)-acetoin strain VJ017 (Fig. 2), which could be due to limitations in NAD^+^ regeneration. For VJ017, NOX activity is required for regenerating two molecules of NAD^+^ for each glucose molecule metabolized, whereas for the strains VJ018 and mBD001 only one NAD^+^ needs to be regenerated, and this apparently enhances glucose utilization. The maximum titers of (3*R*)-acetoin, R-BDO and m-BDO obtained in this study were 27, 43 and 51 g/L respectively and the yield achieved was around 78 to 89% of theoretical maximum. The yield of 2,3-BDO achieved in this study for *L. lactis* was higher than obtained by Gaspar *et al*.^22^ (67% of theoretical maximum), where formate and ethanol were additional by-products.

**Figure 4:**
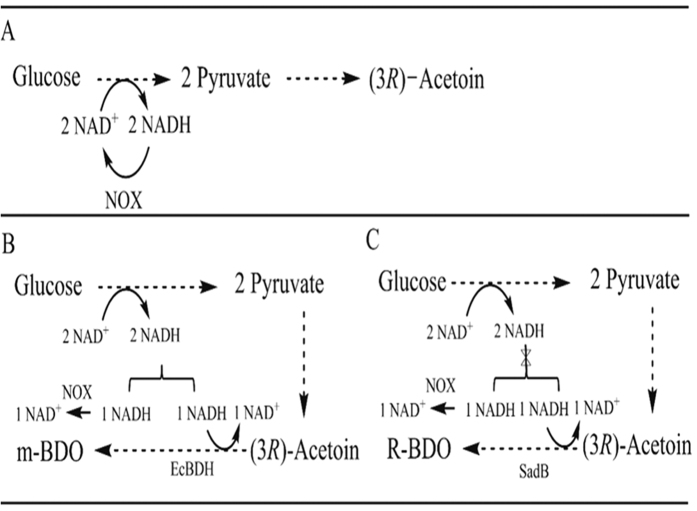
Cofactor regeneration in various *L. lactis* strains. (**A**) Redox balance is achieved by NOX activity during acetoin production with the strain VJ017, (**B**) Co-factor partitioning between NOX and EcBDH during m-BDO production with the strain mBD001, (**C**) Fluctuation in co-factor partitioning between NOX and SadB during R-BDO production with strain VJ018.

Unlike the R-BDO strain VJ018, mBD001 did not produce any detectable amounts of (3*R*)-acetoin throughout the fermentation, which indicates that the cofactor partitioning between NOX and EcBDH is efficient. Ideally, NOX and EcBDH each have to regenerate one NAD^+^ per mole of glucose consumed. Excess NAD^+^ regeneration by NOX could cause formation of the more oxidized product (3*R*)-acetoin (Fig. 4). Lack of (3*R*)-acetoin formation during m-BDO fermentation is most likely due to the highly efficient EcBDH (Figs 2B and 3B). In an earlier study EcBDH was demonstrated to be the most efficient enzyme for 2,3-BDO production in recombinant *E. coli* when compared to other BDHs from native producers *like K. pneumoniae, B. subtilis, S. marcesens* and *B. licheniformis*^23^. Similarly by screening for efficient (2*R*,3*R*)-BDHs from different microorganisms it should be possible to avoid (3*R*)-acetoin formation entirely during R-BDO fermentation. Alternatively, since (3*R*)-acetoin formation indicates excess NAD recycling via NOX activity, one solution could be to limit aeration. Indeed previous reports have shown that the NOX activity can be varied from 0.16 to 0.6 U/mg when the aeration level is varied, and this has an influence on the intracellular availability of NADH^27^,^28^. In this study when we limited the aeration level by decreasing flask to medium ratio, the (3*R*)-acetoin yield (0.044 g/g lactose) was significantly reduced by a factor of approximately 4.5 (Fig. 3C). Interestingly, the availability of glucose also seemed to be critical to achieve a condition that prevents oxidation of R-BDO to (3*R*)-acetoin (Fig. 2C). We observed that as soon as glucose had been depleted, R-BDO was oxidized, and this probably provided the NADH needed for NOX to maintain a reduced intracellular environment and thereby prevent unnecessary oxidative stress. 2,3-BDO oxidation upon substrate exhaustion has also been observed in previous studies^37^.

We also successfully demonstrated the potential of *L. lactis* for converting processed whey waste streams into value added products by using RWP for making (3*R*)-acetoin and BDO isomers. The final biomass obtained (OD_600_ = 10 to 16) was comparable to that obtained with M17 medium with glucose (Figs 2 and 3). The maximum (3*R*)-acetoin, R-BDO and m-BDO yield reached were 0.42, 0.47 and 0.40 g/g lactose with productivities of 0.64, 1.46 and 0.62 g/L·h respectively. The m-BDO productivity was comparable to that obtained using native 2,3-BDO producers like *K. oxytoca*^38^. RWP is a cheap carbon source, and for this study we also added YE as a nitrogen source. We have however previously demonstrated that cheap corn steep liquor hydrolysate can be used to replace YE and thus further lower the medium costs^13^.

Summing up, we have engineered *L. lactis* to efficiently produce the valuable chemicals (3*R*)-acetoin, R-BDO and m-BDO. High yield and product titres were obtained using an abundant and cheap substrate, RWP, and this encourages further optimization of the bioprocess using these strains.

## Materials and Methods

### Strains and plasmids

MG1363 is a plasmid-free derivative of *L. lactis* subsp. *cremoris* strain NCD0712 and other derivatives of MG1363 used for this study are described in Table 3. The plasmid pCS4518 and pTD6 were used to express SadB and EcBDH respectively. For cloning purposes *E. coli* strain Top10 (Invitrogen) {F- *mcrA* Δ(*mrr-hsd*RMS-*mcr*BC) Φ80*lac*ZΔM15 Δ *lac*X74 *rec*A1 *ara*D139 Δ(*araleu*)7697 *gal*U *gal*K *rps*L (StrR) *end*A1 *nup*G} was used. *E. coli* strains were grown aerobically at 37 °C either in Luria-Bertani broth/agar^39^. For growth, MG1363 and its derivatives were cultured in M17 broth/agar supplemented with glucose at 30 °C. When required, antibiotics were added in the following concentrations: erythromycin: 200 μg/ml for *E. coli* and 5 μg/ml for *L. lactis*, tetracycline: 8 μg/ml for *E. coli* and 5 μg/ml for *L. lactis*, chloramphenicol: 20 μg/ml for *E. coli* and 5 μg/ml for *L. lactis*.

### DNA techniques

All manipulations were performed according to Sambrook *et al*.^39^. PfuX7 polymerase was used for PCR applications. Electrocompetent cells of *L. lactis* were prepared by growing in GM17 medium with 1% glycine and transformed by electroporation as described previously. Chromosomal DNA from *L. lactis* was isolated using method described for *E. coli* with the modification that cells were treated with 20 μg of lysozyme per ml for 2 h before lysis. The plasmid vector pCS1966^40^ was used for deleting genes in *L. lactis*. Gene inactivation was achieved by deleting part of the gene, briefly 800-bp regions upstream and downstream of the target to be deleted were PCR amplified and inserted into pCS1966. The resulting plasmids were used as described previously^24^.

### Construction of strains

Derivatives of pCS1966 were used for inactivating *butBA* genes from CS4363, thus giving rise to VJ017. For producing R-BDO, a codon optimized *sadB* gene was amplified using the primers 5′-CTATGTCGACAAGTAATAAAATATTCGGAGGAATTTTGAAATGAAAGCATTAGTATATCATGGAG-3′and 5′-TCATCTGCAGTTATGCTGCTCCTGCATTACTAAG-3′ and gapB promoter from *L. lactis* was amplified was using the primers 5′-TCATGGTACCGAATAAAAATTACTGACAGCCTGC-3′ and 5′-TCAGTC TAGATAGTAGTTTCCTCCTTATAGGGATTAG-3′. The PCR products were further cloned at SalI/PstI and KpnI/XbaI sites of pCS4518 respectively resulting in the plasmid pVJ013. pVJ013 was further transformed to *L. lactis* strains MG1363, VJ017 (MG1363 Δ^3^*ldh* Δ*pta*Δ*adhE*Δ*butBA*) to generate VJ021 and VJ018 respectively. Further the plasmid pJM001 encoding EcBDH driven by high strength synthetic promoter^24^ was transformed into VJ017 to generate mBD001. The lactose plasmid, pLP712 (55.395 kb), was isolated from *L. lactis* NCD0712 was transformed to VJ017, VJ018 and mBD001 and selected in minimal media (SAL) containing lactose as sole carbon source to generate AL002, VJ031 and mL001 respectively.

### Enzyme activity analysis

Cell extract for enzyme assay was prepared as described previously^24^. Briefly, cells were harvested at exponential phase and washed with 0.2% KCl, resuspended in extract buffer and disrupted by glass beads (106-μm diameter; Sigma, Prod. No. G4649) using a FastPrep (MP Biomedicals, Santa Ana, USA). The SadB was assayed for 2,3-butanediol dehydrogenase activity using stereospecific R-BDO as substrate. The assay mixture contained 50 mM potassium phosphate, pH 7.0, and 0.2 mM NAD and cell free extract. The reaction was started by adding 0.2 M R-BDO and formation of NADH was monitored by measuring absorbance at 340 nm using the Infinite® M1000 PRO microplate reader (TECAN) and the accompanying software Magellan.

### Metabolite analysis

Biomass density was measured at OD600 and samples were analysed by high-pressure liquid chromatography to determine product formation and glycolytic flux. HPLC analysis of the fermentation broth was carried out on an Ultimate 3000 high-pressure liquid chromatography system (Dionex, Sunnyvale, CA) equipped with an Aminex HPX-87H column (Bio-Rad, Hercules, CA) and a Shodex RI-101 detector (Showa Denko K.K., Tokyo, Japan). The column oven temperature was set at 60 °C, the mobile phase was 5 mM H_2_SO_4_, and the flow rate was 0.5 ml/min.

### Tolerance studies

To study tolerance of *L. lactis* to acetoin and 2,3-BDO, the strain MG1363 was grown in M17 medium supplemented with 1% glucose at various initial concentration of acetoin and 2,3-BDO. Further, decrease in growth rate was calculated by taking specific growth rate of *L. lactis* without acetoin and 2,3-BDO as 100% using the formula


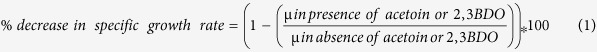


### Fermentation conditions

For acetoin and 2,3-BDO production, the strains VJ017, VJ018 and mBD001 were grown in 250 ml conical flasks with 50 ml M17 broth supplemented with glucose. The cultivation was performed at 30 °C and 200 rpm. Alternatively, acetoin and 2,3-BDO fermentation with VJ031, AL002 and mL001 was performed using residual whey permeate (RWP). 30 ml of diluted RWP containing lactose and 2% (w/v) yeast extract in 250 ml conical flasks was used and the strains were grown aerobically at 30 °C and 200 rpm. Samples were collected periodically to analyze for biomass growth and metabolite formation.

## Additional Information

**How to cite this article**: Kandasamy, V. *et al*. Synthesis of (3*R*)-acetoin and 2,3-butanediol isomers by metabolically engineered *Lactococcus lactis*. *Sci. Rep.* **6**, 36769; doi: 10.1038/srep36769 (2016).

**Publisher’s note:** Springer Nature remains neutral with regard to jurisdictional claims in published maps and institutional affiliations.

## Supplementary Material

Supplementary Information

## Figures and Tables

**Figure 1:**
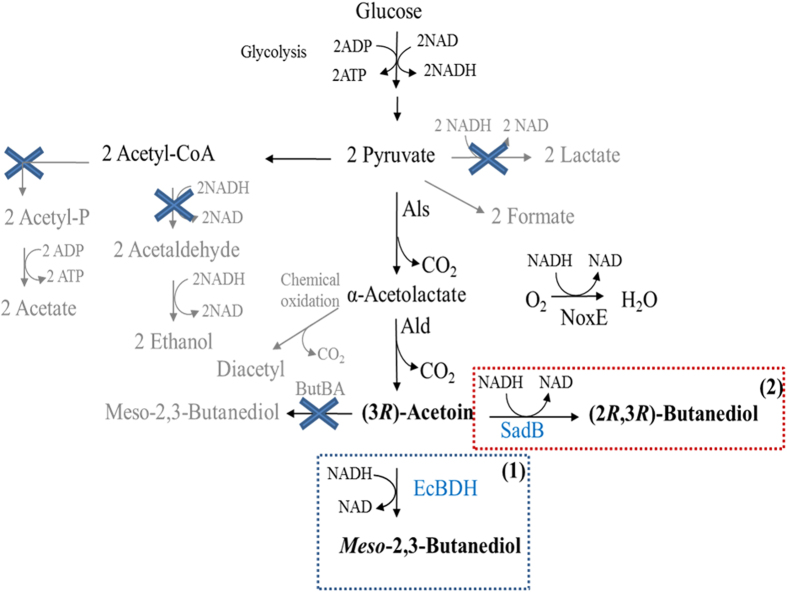
Rewiring the metabolic pathway for synthesis of (3*R*)-Acetoin, R-BDO and m-BDO in *L. lactis*. Als (α-acetolactate synthase); Ald, (α-acetolactate decarboxylase); NoxE, (NADH oxidase); EcBDH (butandediol dehydrogenase from *E. cloacae*; SadB (alcohol dehydrogenase from *A. xylosooxidans*). The blue crosses indicate enzyme activities that have been eliminated. The final product obtained depends on the enzyme activities present, where (3*R*)-acetoin is formed when no additional enzyme activities are introduced, and m-BDO and R-BDO are formed when EcBDH and SadB are heterologously expressed respectively.

**Table 1:** Effect of 2,3-butanediol and acetoin on the growth of *L. lactis* MG1363 in M17 medium supplemented with 1% glucose.

Concentration (g/L)	2,3-Butanediol				Acetoin			
	Specific growth rate (h^−1^)		Decrease in specific growth rate (%)		Specific growth rate (h^−1^)		Decrease in specific growth rate (%)	
	Anaerobic	Aerobic	Anaerobic	Aerobic	Anaerobic	Aerobic	Anaerobic	Aerobic
0	1.17 ± 0.03	1.17 ± 0.01	0	0	1.17 ± 0.03	1.17 ± 0.01	0	0
20	1.1 ± 0.01	0.8 ± 0.01	6	32	1.03 ± 0.02	0.62 ± 0.02	12	47
40	0.97 ± 0.01	0.71 ± 0.01	17	39	0.57 ± 0.01	0.22 ± 0.02	51	81
60	0.82 ± 0.01	0.59 ± 0.01	30	50	0.25 ± 0.01	0.05 ± 0.01	79	96
80	0.68 ± 0.02	0.37 ± 0.02	42	68	0.05 ± 0.01	ND	96	ND
100	0.42 ± 0.03	0.26 ± 0.02	64	78	ND	ND	ND	ND

Values are averages of two independent experiments. ND: not detected. ± Indicates standard deviation from the mean. Decrease in specific growth rate was calculated using equation (1).

**Table 2:** Comparison of product yields of various *L. lactis* strains.

Strains	Growth condition	Yield of fermentation products (g/g)			
		Lactate	Ac	R-BDO	m-BDO
MG1363	Anaerobic	0.9 ± 0.06	ND^a^	ND	ND
CS4363	Aerobic	ND	0.39 ± 0.001	ND	ND
VJ017	Aerobic	ND	0.41 ± 0.001	ND	ND
VJ018^b^	Aerobic	ND	ND	0.41 ± 0.003	ND
mBD001^c^	Aerobic	ND	ND	ND	0.40 ± 0.002

Fermentations were carried out in M17 medium with 1% glucose and products were measured at 24 h. MG1363 was grown under anaerobic conditions whereas CS4363 and VJ017 were grown under aerobic conditions.

^a^ND: Not Detected.

^b,c^Yield was calculated from the fermentation experiment shown in Fig. 2.

Data are means ± standard deviation from two independent experiments.

**Table 3:** Strains and plasmids.

Designation	Genotype or description	Reference
*L. lactis* strains		
CS4363	MG1363 Δ^*3*^*ldh*Δ*pta*Δ*adhE*	20
VJ021	MG1363 pVJ013	This work
VJ017	MG1363 Δ^*3*^*ldh*Δ*pta*Δ*adhE*Δ*butBA*	This work
VJ018	MG1363 Δ^*3*^*ldh*Δ*pta*Δ*adhE*Δ*butBA* pVJ013(pCS45168::*sadB*)	This work
VJ031	MG1363 Δ^*3*^*ldh*Δ*pta*Δ*adhE*Δ*butBA* pVJ013 pLP712	This work
mBD001	MG1363 Δ^*3*^*ldh*Δ*pta*Δ*adhE*Δ*butBA* pJM001	This work
AL002	MG1363 Δ^*3*^*ldh*Δ*pta*Δ*adhE*Δ*butBA* pLP712	This work
mL001	MG1363 Δ^*3*^*ldh*Δ*pta*Δ*adhE*Δ*butBA* pJM001 pLP712	This work
Plasmids		
pCS1966	The selection/counter selection vector	40
pCS4518	pCI372::*gusA*	This work
pVJ013	pCS4518::*sadB*	This work
pJM001	pTD6::FP-*bdh*	24
