# Supplementary material for: Synthesis of (3R)-acetoin and 2,3-butanediol isomers by metabolically engineered Lactococcus lactis
Source: Sci Rep. 2016 Nov 18;6:36769. doi: 10.1038/srep36769 (PMC5114678; doi:10.1038/srep36769)
Supplement: Supplementary Information [file srep36769-s1.doc]

**SUPPLEMENTARY INFORMATION**

**Synthesis of (3*R*)-acetoin and 2,3-butanediol isomers by metabolically engineered *Lactococcus lactis***

Vijayalakshmi Kandasamy^1^,*, Jianming Liu^1^,*, Shruti Harnal Dantoft^1^, Christian Solem^1^, Peter Ruhdal Jensen^1^

^1^National Food Institute, Technical University of Denmark, DK-2800 Kgs. Lyngby, Denmark.

Correspondence and requests for materials should be addressed to P.R.J. (email: perj@food.dtu.dk) or C.S. (chso@food.dtu.dk)

*these authors contributed equally to this work

**Materials and Methods**

**Gas chromatography (GC) analysis**

GC analysis was used to differentiate (3*S*)-acetoin and (3*R*)-acetoin. For this purpose an Agilent 6890 instrument equipped with a chiral column (Supelco β-DEX^TM^ 120, 30 m length, 0.25 mm inner diameter) was applied, where the integrated flame ionization detector was used for the analysis. The carrier gas used was helium (0.8 ml/min). The column oven was maintained at 40^o^C for 1.5 min, then raised to 180^o^C at a rate of 5^o^C/min and then held at 180^o^C for 5 min. In terms of sample preparation, one volume of fermentation broth was extracted with one volume of ethyl acetate and the injection volume was 1 μl with a split ratio of 5:1. For separating the (2*S*,3*S*)-butanediol and (2*R*,3*R*)-butanediol isomers, the same GC column was used, however the conditions were slightly modified. The carrier gas flow was increased to 1.3 ml/min. In terms of temperature profile, the column oven was maintained at 50^o^C for 1.5 min, then raised to 180^o^C at a rate of 8.8^o^C/min and then held at 180^o^C for 5 min. The same procedure was used for sample preparation and the injection volume was 1 μl.


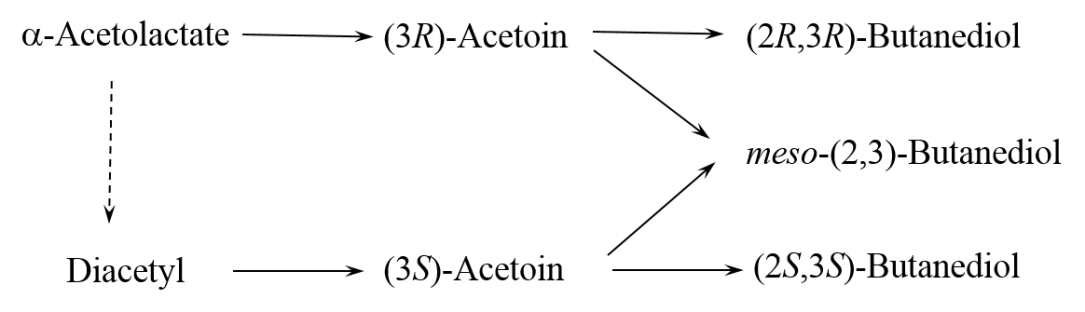


**Figure S1**. The metabolic pathways leading to Acetoin and (2,3)-Butanediol isomers. The reaction from α-acetolactate to diacetyl (dashed line) is a non-enzymatic oxidative decarboxylation.


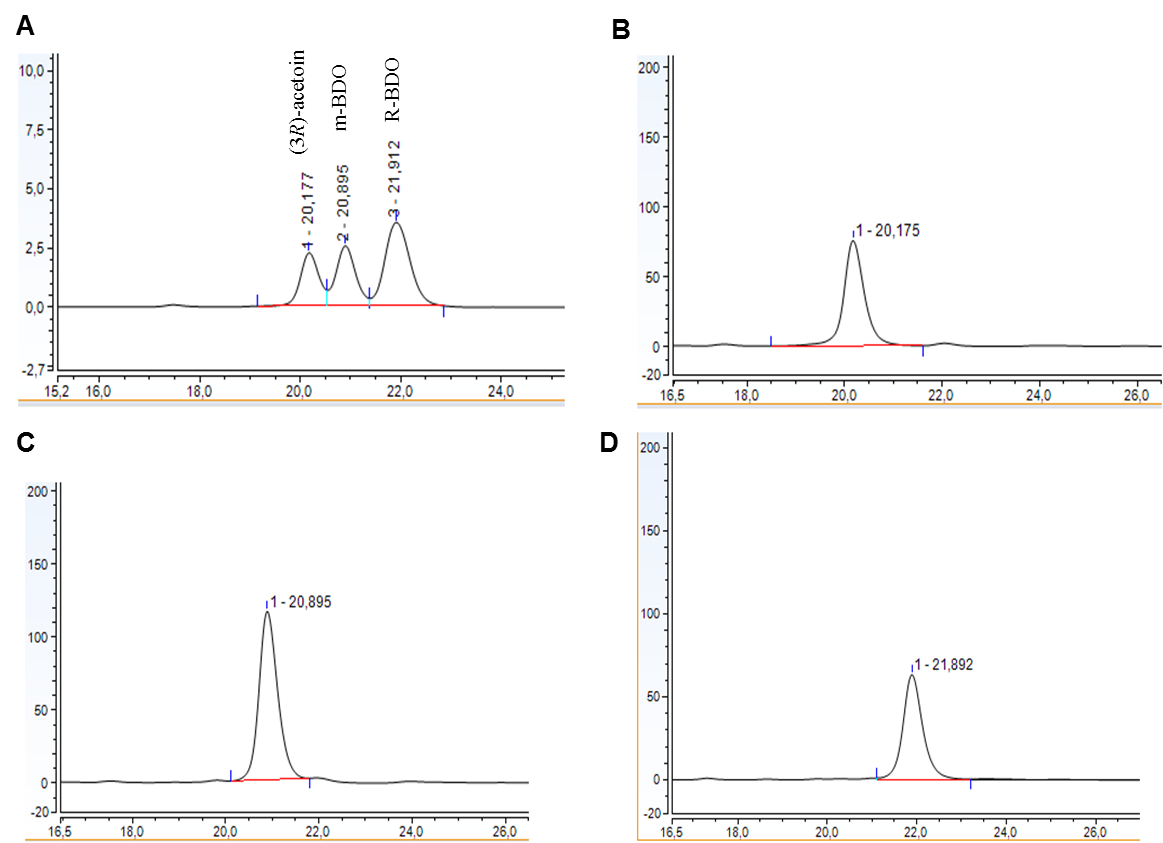


Figure S2. HPLC chromatograms for different samples containing either (3*R*)-acetoin (R-acetoin), *meso*-(2,3)-butanediol (m-BDO), (2*R*,3*R*)-butanediol (R-BDO) or a mixture of these. **A**. standard containing R-acetoin, m-BDO and R-BDO. **B**. detection of R-acetoin in the spent broth of strain AL002. **C**. detection of m-BDO in the spent broth of strain of mL001. **D**. detection of R-BDO in the spent broth of strain of VJ031. The optical isomers of acetoin ((3*R*)-acetoin and (3*S*)-acetoin) and of 2,3-BDO (R-BDO and S-BDO) cannot be separated by HPLC (non-chiral column used), and for this purpose we applied gas chromatography and a chiral column.


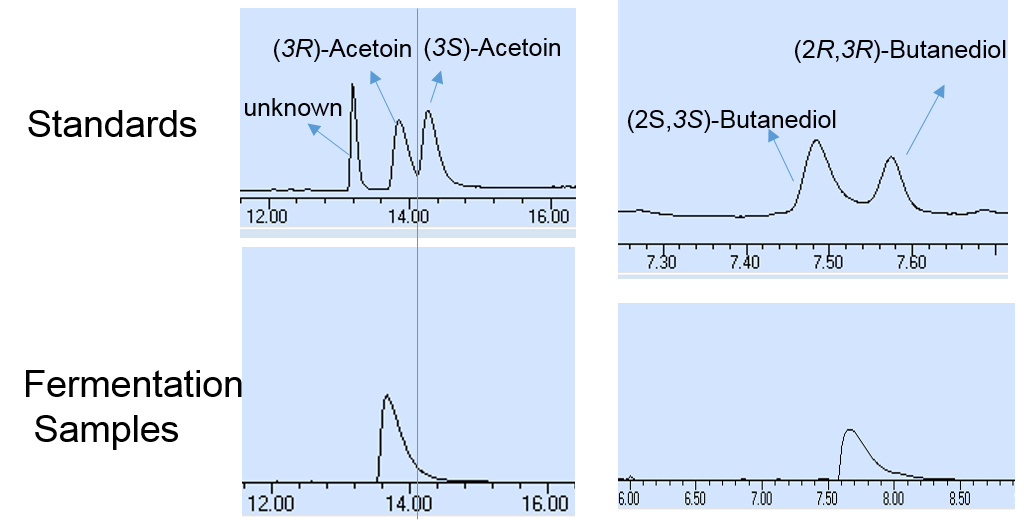


Figure S3. Detection of (3*R*)-acetoin and (2*R*,3*R*)-butanediol in the fermentation broth using gas chromatography. The upper row represents standards containing mixtures of either (3*S*)-acetoin and (3*R*)-acetoin, or (2*S*,3*S*)-butanediol and (2*R*,3*R*)-butanediol. The lower row show the actual chromatograms obtained after analysis of fermentation samples, extracted with equal volume of ethyl acetate.
